# Assessing a possible vulnerability to dental caries in individuals with rare genetic diseases that affect the skeletal development

**DOI:** 10.1186/s13023-019-1114-5

**Published:** 2019-06-18

**Authors:** Heloisa Vieira Prado, Natália Cristina Ruy Carneiro, Matheus França Perazzo, Mauro Henrique Nogueira Guimarães de Abreu, Carolina de Castro Martins, Ana Cristina Borges-Oliveira

**Affiliations:** 1 Department of Dental Public Health, Faculty of Dentistry, Universidade Federal de Minas Gerais, Av. Antônio Carlos, 6627. Campus Pampulha /, Belo Horizonte, MG 31270-901 Brazil; 2 Department of Pediatric Dentistry and Orthodontics, Faculty of Dentistry, Universidade Federal de Minas Gerais, Belo Horizonte, Brazil

**Keywords:** Rare diseases, Genetic diseases, Disabled persons, Oral health, Dental caries

## Abstract

**Background:**

Individuals diagnosed with a rare genetic disease that affects skeletal development often have physical limitations and orofacial problems that exert an impact on oral health. The aim of the present study was to analyze the possible vulnerability to dental caries in individuals with rare genetic diseases that affect skeletal development.

**Methods:**

A paired cross-sectional study was carried out with a sample of 140 individuals [70 with rare genetic diseases affecting skeletal development: mucopolysaccharidosis (MPS) (*n* = 29) and osteogenesis imperfecta (OI) (*n* = 41) and 70 without rare diseases] and their parents/caregivers. The participants in the first group were recruited from two reference hospitals specialized in rare genetic diseases in the city of Belo Horizonte, Brazil. All participants were examined for the evaluation of breathing type, malocclusion, dental anomalies, oral hygiene and dental caries. The parents/caregivers answered a structured questionnaire addressing the individual/behavioral characteristics and medical/dental history of the participants. Statistical analysis involved the chi-square test and multiple logistic regression analysis for the dependent variable (dental caries) (α = 5%). This study received approval from the Human Research Ethics Committee of the Universidade Federal de Minas Gerais.

**Results:**

The mean age of the individuals was 10.34 ± 6.55 years (median: 9.50 years). Individuals with inadequate oral hygiene were 4.70–fold more likely to have dental caries (95% CI: 2.13–10.40) and those with the rare genetic diseases (MPS/OI) were 2.92-fold more likely to have dental caries (95% CI: 1.38–6.17).

**Conclusion:**

Individuals with inadequate oral hygiene and those with MPS and OI had a greater chance of belonging to the group with dental caries. Based on the present findings, individuals with the rare genetic diseases may be considered vulnerable to caries.

## Background

The World Health Organization (WHO) defines rare diseases as all diseases for which the prevalence is less than 65 cases per 100,000 inhabitants [1]. Rare diseases are characterized as debilitating and chronically degenerative and require continuous medical follow up. Affected individuals often have impaired physical, mental, sensorial and behavioral capacities, which can compromise their autonomy with regard to performing activities of daily living [2–6].

Mucopolysaccharidoses (MPS) and osteogenesis imperfecta (OI) are two rare genetic diseases that compromise skeletal development and affect general health. The two diseases lead to dental problems. Studies show that malocclusion, tooth agenesis, tooth rotation and microdontia are common in this population. These diseases are also associated with alterations in genes that regulate the formation of tooth enamel and dentin. Indeed developmental defects of enamel (DDE) are common in individuals with MPS and both dentinogenesis imperfecta (DI) and DDE are common in individuals with OI [7–11].

These dental problems make oral hygiene more difficult [4, 7–9, 12–14]. The low mineral content in dental tissues in interaction with environmental factors may favor the occurrence of dental caries [15, 16]. Moreover, studies have suggested that access to dental services is more difficult for individuals with special needs [9, 10, 17–20]. The difficulty in adequately performing oral hygiene due to the limitations imposed by disease and a lack of information on the part of parents or caregivers about the importance of oral health care can place individuals with special needs in a vulnerable position with regard to dental caries [10, 12, 13, 15, 18–21].

The concept of vulnerability in health is based on the understanding of susceptibility to illness [12, 22, 23]. Illness is considered to arise from a set of individual, collective and contextual factors. This concept also involves the potential for coping with health problems in order to promote strategies for healthcare actions [17, 23]. In the present study, vulnerability is approached from the perspective of the expanded concept of health, exploring the more complex factors of biopsychosocial frailty that expose individuals with rare diseases to dental caries. The investigation of vulnerability provides more integrated means of assessing dental care needs taking into account the abstract and subjective elements associated with the process of becoming ill [4, 21, 23].

Therefore, the aim of the present study was to analyze the possible vulnerability to dental caries in individuals diagnosed with rare genetic diseases that affect skeletal development.

## Methods

A paired cross-sectional study was carried out with a sample of 140 individuals (70 with rare diseases and 70 without rare diseases) between two and 27 years of age and their parents/caregivers. A convenience sample was selected of individuals with two rare genetic diseases affecting skeletal development: MPS (*n* = 29) and OI (*n* = 41).

The group with rare diseases was recruited from two public hospitals in the city of Belo Horizonte, Brazil. The hospitals are reference centers for the treatment of these two diseases. Individuals without rare diseases were recruited from outpatient clinics at the same two hospitals. All hospitals belong to the public healthcare system. This study received from the Human Research Ethics Committee of the Universidade Federal de Minas Gerais (certificate numbers: 01480212.4.0000.5149 [MPS] and 03027612.7.000.5149 [OI]).

The PS program (Power and Sample Size Calculation, version 3.0, Nashville, TN, USA) was used to calculate the test power. Considering the data obtained, the probability of exposure to dental caries among the controls was 34.3% and the correlation coefficient for exposure between matched cases and controls was 0.5. The odds ratio for dental caries among the individuals with rare diseases compared to the control group was 4.1. Thus, the test power was 100%, with a 5% margin of type I error.

### Data collection

Data collection involved oral examinations of the participants and the administration of a questionnaire to parents/caregivers addressing sociodemographic and behavioral aspects of the participants (based on Oliveira et al., 2008a [17]; 2008b [24]). The type of rare disease was identified by the patient’s medical record. Economic status was determined based on the Brazilian Economic Classification Criteria (ABEP), which addresses the purchasing power and general situation of households and classifies families in A1 (highest), B1, B2, C1, C2, D and E (lowest). Classes were categorized into high (A1, B1 and B2), middle (C1 and C2) and low (D and E) [25]. Ethnicity was categorized using the criteria established by the Brazilian Institute of Geography and Statistics (IBGE) for skin color: white, black, brown or yellow [26].

The oral examinations were performed by two examiners with the patient sitting in a chair under artificial light (Petzl Zoom head lamp®, Petzl America, Clearfield, UT, USA). The examiners used a mouth mirror (Duflex® n°5), Community Periodontal Index probe (Golgran®, São Paulo, SP, Brazil) and appropriate personal protective equipment to avoid cross infection. Radiography was not employed.

Breathing type was determined using the oral mirror test. A double-faced oral mirror was placed under the patient’s nose. If the patient was a mouth breather (either alone or with nasal breathing), the mirror would be fogged on the lower portion; if the patient had only nasal breathing, the mirror would be fogged only on the upper portion [24, 27].

The following malocclusions were investigated: overjet (increased/protrusion, anterior crossbite, absent), overbite (increased/deep bite, anterior open bite, absent, top) and posterior crossbite. The following dental anomalies were investigated: conical tooth, tooth agenesis, tooth rotation, DDE and DI. Tooth agenesis was considered a possible diagnosis, because the oral examination was performed only clinically. The diagnostic criteria for malocclusion and dental anomalies were based on Seow (2014) [28], Oliveira et al. (2008b) [24] and the WHO (2013b) [29].

The Simplified Oral Hygiene Index (OHI-S) was used to evaluate oral hygiene and was scored as follows: 0 = Absence of dental plaque/dental calculus; 1 = little dental plaque/dental calculus, less than 1/3 of dental surface covered; 2 = dental plaque / dental calculus covering more than 1/3 and less than 2/3 of the dental surface; and 3 = dental plaque/dental calculus covering more than 2/3 of dental surface. Plaque and dental calculus were evaluated separately [17, 21, 30]. The final result of the OHI-S was obtained from the sum of the codes divided by the total number of teeth examined and classified as satisfactory (0 to 1), fair (1.1 to 2), deficient (2.1 to 3) or poor (≥3.1). The classification was dichotomized as adequate (satisfactory and fair) or inadequate (deficient and poor).

Dental caries was assessed according to the WHO diagnostic criteria [29]. The number of decayed (presence of cavitated lesion) primary and permanent teeth was recorded.

### Training and calibration process

Training and calibration exercises were performed prior to the main study and were divided into theoretical and practical steps. The theoretical step involved the analysis of images of malocclusions, dental anomalies, different levels of oral hygiene and dental caries. The practical step performed at one of the hospitals selected for the main study. Due to the limited number of individuals with MPS and OI, only individuals without rare diseases were examined during the calibration process. These individuals were not included in the final sample. The results of the examiners were compared to the findings of an experienced epidemiologist (gold standard) using the Kappa statistic. Agreement was very good, with Kappa coefficients between 0.76 and 0.98 for all conditions examined.

### Pilot study

A pilot study was carried out after the calibration process involving 10 individuals with rare diseases and their respective parents/caregivers at the previously selected public hospitals. The pilot study indicated that no changes to the methodology were required. The participants in the pilot study were included in the main study.

### Directed acyclic graph

Prior to the data analysis, a directed acyclic graph (DAG) was used to select the covariates for the statistical adjustments. This is a theoretical method with visual representations of causal assumptions that is increasingly used in modern epidemiology to help identify confounding factors for the causal question at hand [31].

**Fig. 1:**
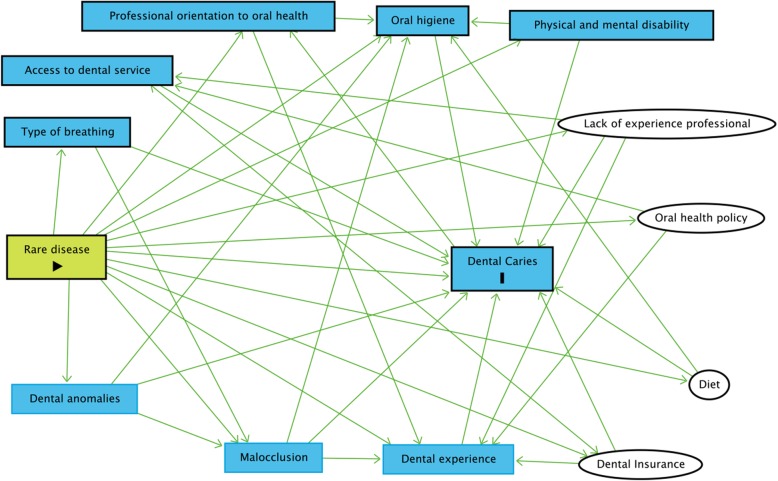
Directed acyclic graphs (DAG)

To identify possible confounding variables in the association between the rare diseases and dental caries, individual factors (diet, oral hygiene, physical and mental disability) [13, 18, 20, 21, 32] and clinical factors (malocclusion, dental anomalies and breathing type) [11, 24, 33, 34] were included in the DAG model. Variables related to contextual and collective factors (previous dental experience, professional advice to consult a dentist, access to dental services, oral health policies, lack of experienced professionals and dental insurance) [11, 17, 19, 35] were also included. Based on the model, there were no confounding factors in the association between the rare diseases and dental caries. As the individuals in the different groups were matched for age, sex and economic status, these variables were not incorporated into the DAG (Fig. 1).

### Statistical analysis

Statistical analysis was performed using the Statistical Package for the Social Sciences (SPSS for Windows, version 21.0, IBM Inc., Amonk, NY, USA). The chi-square test was used to determine the association between the exposure (dental caries) and the independent variables (*p* < 0.05). Multiple logistic regression analysis was performed to identify the independent impact of each variable studied. The independent variables were included in the decreasing logistic model in accordance with their statistical significance (*p* < 0.25; backward stepwise procedure).

## Results

The age of the 140 subjects examined ranged from two to 27 years (mean: 10.34 ± 6.55 years; median 9.50 years). The mean age of the parents/caregivers was 37.93 ± 9.00 years (median: 37.00 years).

**Fig. 2:**
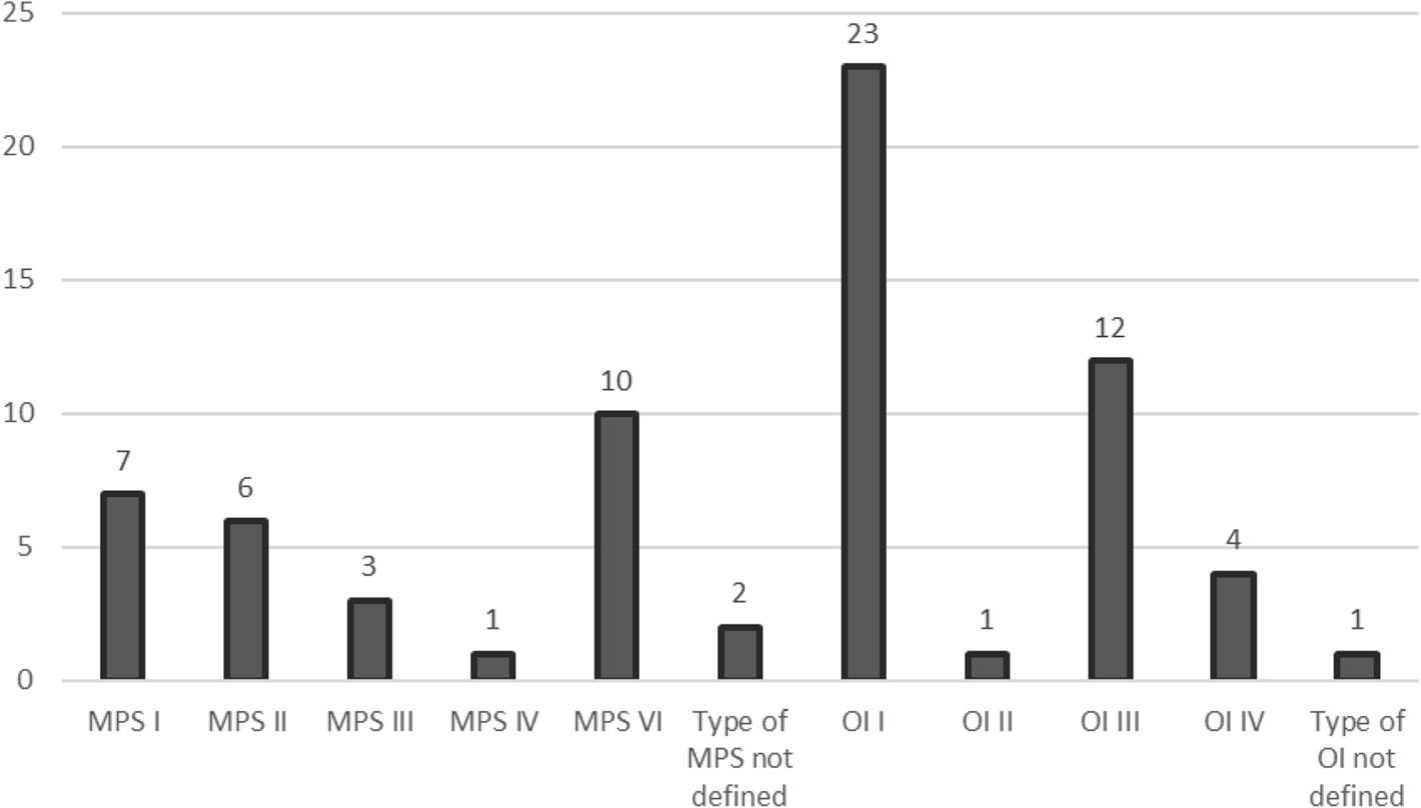
Distribution of sample according type of rare disease (*n* = 70)

**Fig. 3:**
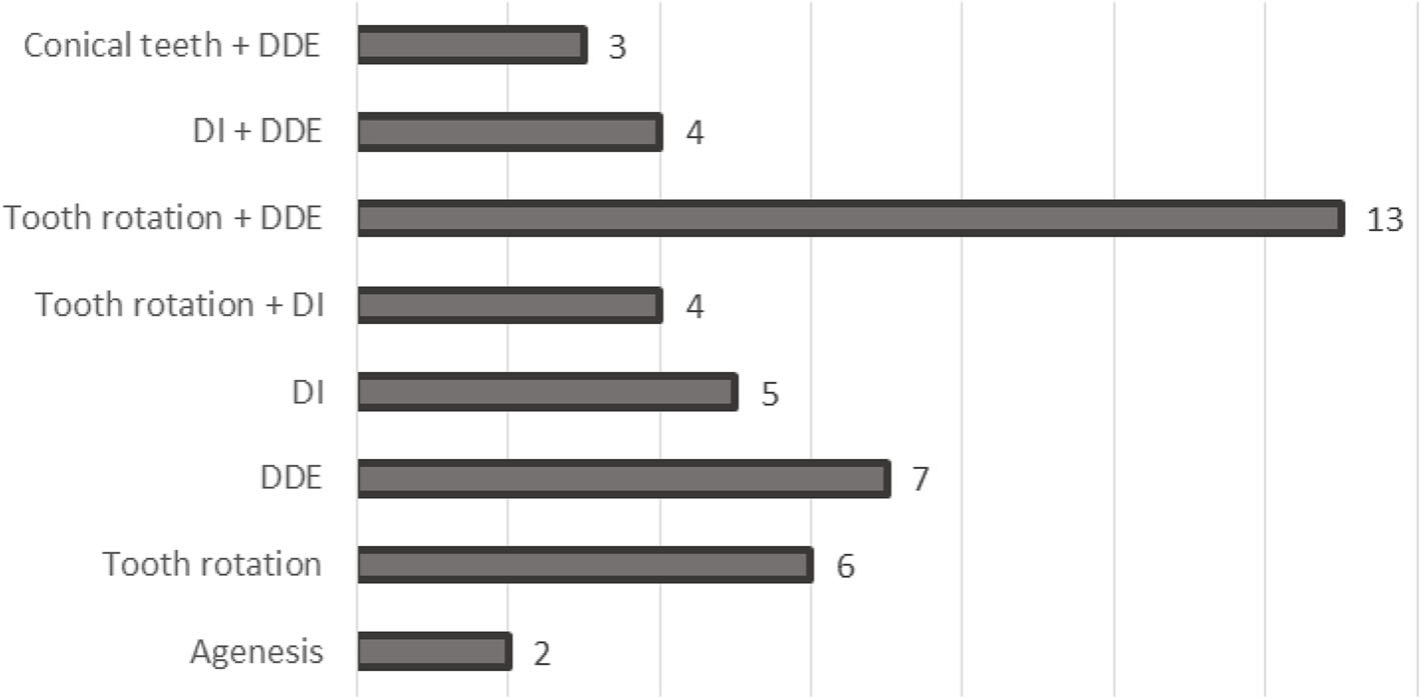
Distribution of sample according type of dental anomaly (*n* = 44). DDE = developmental defects of enamel. DI = dentinogenesis imperfecta

The distribution of the 70 individuals with rare diseases is represented in Fig. 2. The most frequent types of dental anomalies in the population studied were DDE, DI and tooth rotation (Fig. 3).

No significant differences between groups were found regarding sex (*p* = 1.000), age (*p* = 0.723), skin color (*p* = 0.859), parent’s/caregiver’s schooling (*p* = 0.205) and economic status (*p* = 0.301) (Table 1).

**Table 1:** Distribution of sample of individuals with and without rare diseases (*n* = 140)

Variables	GROUP			*p*-value *
	With rare diseases n (%)	Without rare diseases n (%)	Total n (100%)	
Sex				
Male	42 (49.2)	43 (50.6)	85	1.000
Female	28 (50.9)	27 (49.1)	55	
Age (years)				
2–12	44 (48.4)	47 (51.6)	91	0.723
13–27	26 (53.1)	23 (46.9)	49	
Skin color				
Black / Brown	45 (48.9)	47 (51.1)	92	0.859
White	25 (52.1)	23 (47.9)	48	
Parent’s/caregiver’s education (years)				
≥ 8	44 (46.3)	51 (53.7)	95	0.205
< 8	26 (57.8)	19 (42.2)	45	
Economic class				
High (A + B)	27 (56.3)	21 (43.8)	48	0.301
Middle (C)	40 (48.8)	42 (51.2)	82	
Low (D + E)	3 (30.0)	7 (70.0)	10	

* X^2^ test (5% significance level)

Rare disease (*p* < 0.001) and oral hygiene (*p* < 0.001) were significantly associated with dental caries (Table 2).

**Table 2:** Absolute and relative frequency of sample according to prevalence of dental caries (*n* = 140)

Independent variables	DENTAL CARIES		Total n (100%)	*p*-value*
	Present n (%)	Absent n (%)		
Age (years)				
2–12	46 (50.5)	45 (49.5)	91	0.777
13–27	26 (53.1)	23 (46.9)	49	
Sex				
Male	45 (52.9)	40 (47.1)	85	0.656
Female	27 (49.1)	28 (50.9)	55	
Skin color				
Black / Brown	53 (57.6)	39 (42.4)	92	0.054
White	19 (39.6)	29 (60.4)	48	
Economic class				
Less favored (D + E)	6 (60.0)	4 (40.0)	10	0.544
Favored (C)	44 (53.7)	38 (46.3)	82	
Most favored (A + B)	22 (45.8)	26 (54.2)	48	
Parent’s/caregiver’s education (years)				
< 8	27 (60.0)	18 (40)	45	0.163
≥ 8	45 (47.4)	50 (52.6)	95	
Rare disease				
Present	48 (68.6)	22 (31.4)	70	**< 0.001**
Absent	24 (34.3)	46 (65.7)	70	
Previous dental experience				
No	36 (56.3)	28 (43.8)	64	0.295
Yes	36 (47.4)	40 (52.6)	76	
Professional advice to consult a dentist				
Yes	42 (56.0)	33 (44.0)	75	0.245
No	30 (46.2)	35 (53.8)	65	
Type of breathing				
Oral	17 (58.6)	12 (41.4)	29	0.384
Nasal	55 (49.5)	56 (50.5)	111	
Malocclusion				
Present	52 (52.0)	48 (48.0)	100	0.831
Absent	20 (50.0)	20 (50.0)	40	
Dental anomaly				
Present	27 (61.4)	17 (38.6)	77	0.111
Absent	45 (46.9)	51 (53.1)	96	
Oral hygiene				
Inadequate	41 (75.9)	13 (24.1)	54	**< 0.001**
Adequate	30 (34.9)	56 (65.1)	86	

*X^2^ test (5% significance level) / bold type: statistically significant difference (*p* < 0.05)

Table 3 displays the results of the multiple logistic regression analysis. The variables “oral hygiene” and “rare disease” remained in the final model. Individuals with rare diseases had a 2.92-fold greater chance of belonging to the group diagnosed with dental caries (95% CI: 1.37–6.17; *p* = 0.005) and those with inadequate oral hygiene had a 4.70-fold greater chance of belonging to the group diagnosed with dental caries (95% CI: 2.13–10.40; *p* < 0.001).

**Table 3:** Multiple logistic regression models explaining prevalence of dental caries in individuals with and without rare diseases (*n* = 140)

Dependent variable	Independent variables	OR (95% CI) Crude	OR (95% CI) Adjusted	*p*-value
Dental caries	Oral hygiene (Inadequate)	4.57 (2.06–10.16)	**4.70 (2.13–10.40)**	**< 0.001**
	Rare disease (Present)	2.69 (1.21–5.97)	**2.92 (1.38–6.17)**	**0.005**

*OR* Odds ratio, *CI* Confidence interval

bold type: statistically significant difference (*p* < 0.05)

## Discussion

The vulnerability concept discusses the health-disease process taken in account the more complex causes associated with it [22, 23]. The analysis of vulnerability to dental caries is an important point of reflection for formulating measures to ensure greater protection for vulnerable individuals. In contrast, isolated emergency actions do not modify causality.

The limitations imposed by some rare diseases can negatively impact quality of life [2–6, 9, 12, 14] and the occurrence of oral problems exacerbates this situation. Dental caries can cause acute or chronic pain, the formation of fistulas and abscesses, and tooth loss [17, 19, 20]. The consequences of untreated tooth decay can affect different aspects of life, such as activities of daily living, sleep, speech, eating, social relations and self-esteem [17, 19–21].

The rare genetic diseases MPS and OI were chosen for the present investigation because both affect the development of the skeletal system. Malocclusions and dental anomalies are also commonly found in this population [4, 7–11]. Furthermore, care for the patients of these conditions is offered a university hospital that is considered to be a reference center for the treatment of rare genetic diseases.

In the present study, individuals with a poor oral hygiene (with or without a rare disease) had a greater chance of belonging to the group with dental caries. The individuals with rare diseases were also more likely to belong to the group with dental caries. The influence of oral hygiene on the prevalence of dental caries has been widely discussed [36–38]. The increased chance of individuals with rare diseases having dental caries is the result of a set of factors. According to some authors, not only individual factors, but also collective and contextual factors lead to greater susceptibility to dental caries [22, 23].

A physical limitation or motor impairment in a disabled individual can lead to dependence with regard to performing activities of daily living, such as oral hygiene [2, 12, 13, 21]. Moreover, parents and caregivers often have difficulties with this activity, performing it improperly, infrequently or even not at all [4, 13, 18, 20, 21].

In the present study, the results did not prove that dental anomalies and malocclusion were associated with a greater chance of dental caries vulnerability in individuals with MPS and OI or those without rare genetic diseases. However, is important to consider that previous studies showed that the presence of malocclusion and dental anomalies can lead to the retention of food scraps and the accumulation of dental plaque and can also hamper tooth brushing [7, 8, 10, 12–14].

Depending on its extent and the involvement of the organism, a rare disease can lead to a stressful routine of constant medical appointments, therapies and hospitalizations [2, 5, 6, 9, 12, 14]. As a consequence of this and also due to a lack of information and guidance, parents/caregivers of affected children often fail to prioritize oral health [9, 10, 15, 19, 21], which constitutes a barrier to early and preventive dental care. It is therefore important for the medical team that cares for the patient with a rare disease to advise the parents/guardians to take him/her to the dentist [12, 17, 18, 21, 22].

Previous studies suggest that the lack of oral health policies and programs targeting this portion of the population as well as limited knowledge and experience regarding the peculiarities of rare diseases make many oral health professionals feel uncomfortable and unprepared to treat these individuals [9, 10, 12–14, 19, 33]. This constitutes another barrier to preventive dental care and consequently increases the risk of dental caries among individuals with rare diseases.

Some limitations of the present study should be considered. First, the factors associated with greater vulnerability to dental caries in individuals with MPS and OI were not investigated. Further research is needed to better understand such association. The cross-sectional this study design impedes the inference of causal relationships. Moreover, the use of questionnaires is always accompanied by the risk of recall bias on the part of respondents. However, this study also has strengths that should be highlighted. The use of a matched control group without rare diseases minimizes the possible influence of the matched characteristics on the association between the dependent and independent variables. The authors also used a DAG to identify possible confounding factors and explore the influence of individual, collective and contextual factors on vulnerability to dental caries.

It is certainly of great importance to reflect on vulnerability to dental caries among individuals with rare diseases, since such patients are a small part of the general population and do not enjoy the same visibility in terms of preventive measures implemented by public health programs. The greater commitment to general health care often leads to the neglecting of dental care needs. The lack of referral and orientation from other health professionals can aggravate the oral health status of these individuals, who also occupy an unfavorable position in terms of access to health services and they are less likely to receive dental care compared to the general population. This is presumably due to their physical limitations as well as difficulties oral health professionals face when treating these patients.

The study of vulnerability to dental caries allows theoretical approximations that are not restricted to individual behavior and the biomedical approach. It is necessary to strengthen integral care for individuals with rare diseases, ensuring access to dental services through public policies and professional training. Professionals who treat patients with rare diseases should advise parents and caregivers regarding the importance of caring for their children’s oral health.

## Conclusion

Individuals with inadequate oral hygiene and individuals with MPS and OI had a greater chance of belonging to the group with dental caries. Based on these findings, individuals with the rare genetic diseases studied may be considered vulnerable to caries.

## Data Availability

All data generated and analyzed during this study are included in this published article.

## Abbreviations

DDE: Developmental defects of enamel
DI: Dentinogenesis imperfecta
MPS: Mucopolysaccharidosis
OI: Osteogenesis imperfecta
